# Low‐carbohydrate diet score and risk of bladder cancer: Findings from a prospective cohort study

**DOI:** 10.1002/bco2.70033

**Published:** 2025-06-02

**Authors:** Yen Thi‐Hai Pham, Renwei Wang, Jian‐Min Yuan, Hung N. Luu

**Affiliations:** ^1^ University of Pittsburgh Medical Center Hillman Cancer Center Pittsburgh PA USA; ^2^ Department of Epidemiology, School of Public Health University of Pittsburgh Pittsburgh PA USA; ^3^ Dr. Mary and Ron Neal Cancer Center Houston Methodist Research Institute, Houston Methodist Hospital Houston TX USA

**Keywords:** bladder cancer risk, Low‐carbohydrate diet score, prospective cohort study

## Abstract

**Objective:**

Low carbohydrate diet (LCD), a summary score considering sources of all macronutrients in a dietary pattern, is defined by lower intakes of carbohydrates and higher intakes of proteins and fats. Research on the role of LCD and risk of bladder cancer is scare. We, therefore, prospectively examined the association between LCS scores and bladder cancer risk.

**Patients and Methods:**

We used data from the Singapore Chinese Health Study, a prospective cohort study of 63 275 participants aged 45–74 living in Singapore who were recruited during 1993–1998 period. LCD scores were derived from the semi‐quantitative food frequency questionnaire at baseline. Bladder cancer cases were identified through record linkage with the Singapore cancer registry. Cox proportional hazard regression method was used to calculate hazard ratios (HRs) and 95% confidence intervals (CIs) for bladder cancer in relation with LCD scores.

**Results:**

After 17.6 years of follow‐up with 819 573 person‐years, 250 participants developed bladder cancer. We found a statistically significant, positive association for bladder cancer risk with increasing level of animal‐based LCD (HR_per‐SD increment_ = 1.16, 95% CI: 1.02–1.32; *P*_*trend*_ = 0.01), but a null association with an increased level of plant‐based LCD (HR_per‐SD increment_ = 1.08, 95% CI: 0.91–1.28, *P*_*trend*_ = 0.78).

**Conclusion:**

In summary, we showed that an LCD diet with fat and protein from animal‐based food was associated with increased risk while an LCD diet with fat and protein derived mainly from plant‐based food was not associated with bladder cancer risk. Our findings have implications for diet modifications in the prevention and control program of bladder cancer.

**Abbreviations**
BMI: body mass index
CI: confidence interval
HR: hazard ratio
LCD: low‐carbohydrate diet
SCHS: Singapore Chinese Health Study
SD: standard deviation

## 1 INTRODUCTION

Bladder cancer is a leading malignancy of the urinary tract with annually more than 570 000 new cases and more than 200 000 deaths worldwide.^1^ In the United States, the current incidence rate of bladder cancer is 15.8 per 100 000 population with more than 82 000 new cases and more than 16 000 deaths each year.^2^ The fact that men have four times the incidence rate as that of women^1^ might reflect the difference between men and women in historical smoking prevalence and exposure to occupational carcinogens.^3^ Smoking is an established major risk factor for bladder cancer and accounts 30–50% of bladder cancer cases.^4^ However, identifying factors other than smoking, especially modifiable ones such as dietary factors, can improve our understanding of bladder cancer aetiology and may provide opportunities for bladder cancer prevention.

Low‐carbohydrate diet (LCD) is a dietary pattern to restricts carbohydrates and favours protein and/or fat intakes that has recently been used for weight loss.^5^, ^6^ While its short‐term effect to support weight loss has been recognized,^7^ its long‐term effect on chronic diseases, including cancer, needs to be elucidated. The LCD score is a summary scale to quantify the extent of a low‐carbohydrate diet and has three important elements of the daily diet: 1) carbohydrate, 2) fat and 3) protein,^8^ which evaluates holistically the dietary pattern rather than single nutrients. There are three types of LCD scores: 1) the total (or overall) LCD score represents the summed intake of carbohydrates, fats and proteins from all dietary sources, 2) the animal‐based and 3) plant‐based LCD scores that are derived from animal and plant food sources, respectively.

Different studies have investigated the association between LCD and health outcomes. For instance, we reported that there was a positive association between LCD and risks of hepatocellular carcinoma^9^ and colorectal cancer^10^ in the Chinese Singaporean population. Data from the Japan Public Health Center‐based Prospective Study (JPHC) showed that a higher animal‐based LCD score was associated with a higher risk of overall cancer, colorectal cancer and lung cancer whereas plant‐based LCD score was associated with a lower risk of gastric cancer.^11^ Yet, LCD was found to be inversely associated with risk of pancreatic cancer in the Prostate, Lung, Colorectal, and Ovarian (PLCO) Cancer Screening Trial.^12^ Plant‐based LCD was also reported to be associated with reduced risk of type 2 diabetes among women in the Nurses' Health Study (NHS)^8^ and Japanese men in the Japan Collaborative Cohort Study (JACC) Cohort.^13^ The total LCD score and animal‐based LCD were also reported to be associated with increased risk of type 2 diabetes among Australians in the Melbourne Collaborative Cohort Study (MCCS), which was largely explained by obesity,^14^ among Iranian adults^15^ and U.S. men in the Health Professional Follow‐up Study (HPFS).^16^ A recent analysis from the NHS and HPFS cohorts also showed that among patients with type 2 diabetes, greater adherence to LCD was significantly associated with lower risk of total, cardiovascular and cancer mortality.^17^

However, data on dietary LCD score and risk of bladder cancer are sparse. To date, only one epidemiologic study among the Japanese population examined and found a null association between the LCD scores and the risk of bladder cancer.^11^ The present study investigated the association between LCD scores and bladder cancer risk in another prospective cohort study in Asians, the Singapore Chinese Health Study (SCHS) consisting of more than 63 000 Chinese Singaporeans. We examined separately for total LCD, animal‐based‐ and plant‐based LCD scores with risk of incident bladder cancer and provided additional data on the effects of low‐carbohydrate diet on in the development of bladder cancer in Asian populations.

## 2 PATIENTS AND METHODS

### 2.1 Study population

We used data from the Singapore Chinese Health Study (SCHS) for the current analysis. Details of its protocol and enrollment were described previously.^18^ Briefly, the SCHS is an on‐going population‐based prospective cohort study of 63 257 Chinese men and women, aged 45–74 years old at the baseline. The enrollment occurred during the April 1993 and December 1998 period. Participants of the SCHS were from two main dialect groups of Chinese in Singapore, the Hokkiens and Cantonese who migrated from two southern provinces of China, the Fujian and Guangdong, respectively. The SCHS was conducted in accordance with both the Declarations of Helsinki and Istanbul. All study participants agreed to provide informed consent. The SCHS study has been continuously approved by the Institutional Review Boards (IRBs) of the National University of Singapore and the University of Pittsburgh.

At baseline, trained interviewers conducted home interviews to study participants to collect information on demographics, body weight and height, family history of cancer, lifetime use of tobacco, menstrual/reproductive history (women only), occupational exposure, current physical activity and medical history using a structured questionnaire. Subsequently, train interviewers called participants to obtain updated information on alcohol use, tobacco smoking, medical history, current physical activity and body weight and asked whether they agreed to provide blood and urine samples for research. During the July 1999 and December 2003 period, 28 346 participants (or about 57%) agreed to provide blood for research purposes.

### 2.2 Dietary assessment

We used a semi‐quantitative food frequency questionnaire (FFQ) to collect dietary information from study participants in the SCHS. Details on the FFQ development and validation were described elsewhere.^19^ Briefly, the FFQ used in the SCHS comprised 165 food items or food groups that were commonly consumed by Chinese Singaporeans. We asked study participants how frequently (in eight categories: ranging from “*never or hardly ever*” to “*two or more times a day*”) they consumed the food or food group, followed by a question on the amount of food consumed, using photographs to choose from three portion sizes (i.e., small, medium and large). We used the Singapore Food Composition Database to calculate the average daily intake of approximately 100 nutrients and non‐nutrient compounds for each study participant.^19^ Between April 1994 and March 1997, a validation study of the FFQ was conducted among a random sample of 810 participants of SCHS participants. The FFQ was validated against two 24‐hour dietary recalls (24‐HDRs), one on a weekday and the other on a weekend that was approximately two months apart. The correlation coefficients between the FFQ and 24‐HDR for the majority of calorie‐adjusted nutrients ranged from 0.24 (for saturated fat intake in Hokkien women) to 0.73 (for saturated fat intake in Cantonese men).^19^

### 2.3 Low‐carbohydrate score (LCD) calculation

In the current analysis, we calculated three LCD scores separately—total, animal‐based and plant‐based LCD scores, which were developed and validated in prior works.^8^, ^9^, ^10^, ^20^ Briefly, we first ranked cohort participants by their daily intakes of total calories from carbohydrates, fats and proteins, respectively. We then divided them evenly into 11 groups according to their ranks by each of the three calorie sources, respectively. We assigned each participant in the lowest rank of carbohydrate intake a score of 10 and a subject in the highest rank of carbohydrate intake a zero score. On the contrary, we assigned subjects with the lowest intake of fat and protein a zero and those in the highest rank a score of 10. As a result, the total LCD score was calculated as a sum of the rankings over three different macronutrients, ranging from 0 to 30 and reflecting the highest consumption of protein and fat and the lowest consumption of carbohydrate (Table S1). Similarly, the animal‐based and plant‐based LCD scores were calculated based on the intakes of fat and proteins from animal and plant sources, respectively.

### 2.4 Assessment of other covariates

Body mass index (BMI) was calculated as the weight in kilograms divided by height in meters squared. Smoking status was grouped into never, current and former smokers.^21^ For current and former smokers, we also collected information on number of cigarette per day and number of years of smoking. Alcohol drinking status was collected as non‐drinker, monthly drinker, weekly drinker and daily drinker. For physical activity, we used a continuous scale of eight, including never, 0.5–1, 2–3, 4–6, 7–10, 11–20, 21–30 and 31 hours or more per week, for each of three physical activity categories: 1) strenuous sports (i.e., jogging, bicycling on hills, tennis, squash, swimming laps, or aerobics); 2) vigorous work (i.e., moving heavy furniture, loading or unloading trucks, shovelling, or equivalent manual labour) and 3) moderate activities (i.e., brisk walking, bowling, bicycling on level ground, tai chi and chi kung).^22^ Coffee drinking status was categorized as non‐drinker/monthly/weekly drinker, 1 cup/day, 2–3 cups/day and ≥4 cups/day. History of diabetes was collected by asking participants whether they were told by their doctor that they had diabetes and if so, at what age they had the first diagnosis.

### 2.5 Ascertainment of bladder cancer cases

Deaths and cancer diagnoses among study participants were ascertained through the linkage analysis with the Singapore Registry of Births and Deaths and the Singapore Cancer Registry, respectively. The Singapore Cancer Registry has been comprehensively collected information on cancer diagnosis since 1968.^23^ As of December 31, 2015, only less than 1% of (or 56 participants) were lost to follow‐up due to their migration out of Singapore. In the current analysis, the International Classification of Diseases‐Oncology, 2nd Edition (ICD‐O‐2) code C67.x was used to identify bladder cancer cases. After excluding 1936 participants with a history of cancer at recruitment, the current analysis comprised 250 incident cases of bladder cancer and 61 071 study participants who were free of cancer at baseline.

### 2.6 Statistical analysis

Frequencies were calculated for categorical variables and mean and standard deviation (SD) for continuous variables. We used *t*‐test and *χ* ^*2*^ test to compare the difference in distributions of continuous and categorical variables, respectively, between bladder cancer cases and non‐cases as well as across categories of LCD scores. We defined quartiles of LCD scores based on their distributions among the entire cohort. We calculated person‐years at risk for each participant from the date of baseline interview to the date of diagnosis of bladder cancer, death, migration out of Singapore, or December 31, 2015, whichever occurred first.

In the main analysis, we used the Cox proportional hazard regression model to evaluate the associations between LCD scores and the risk of bladder cancer. We calculated hazard ratios (HRs) and their 95% confidence intervals (CIs) for bladder cancer according to different levels of LCD scores, both in quartiles and continuous scales. In the multivariable Cox regression models, the following covariates were included as potential confounding factors: age at recruitment (years), sex, dialect group (Hokkien or Cantonese), level of education (no formal education, primary school, secondary or higher education), year of enrollment (1993–1995 vs 1996–1998), BMI (kg/m^2^), smoking status (never vs. ever), alcohol intake (non/monthly drinkers, weekly, daily drinkers), coffee drinking status (non‐drinker/monthly/weekly drinker, 1 cup/day, 2–3 cups/day and ≥4 cups/day), history with type 2 diabetes (yes vs. no), physical activity (yes vs. no), total soy intake,^24^ and total energy intake (KCal/day). Study participants with BMI ≥ 23 kg/m^2^ were considered overweight or obese, following the recommendation from the World Health Organization (WHO) for the Asian population.^25^, ^26^

We further conducted stratified analysis by sex (male vs. female), BMI (<23 kg/m^2^ vs. ≥23 mg/m^2^), smoking status (ever vs. never), alcohol drinking status (non/monthly drinker vs. weekly/daily drinker) and history of diabetes (yes vs. no). Because of the exploring nature of stratified analysis, we used Bonferroni correction in which *P‐value* = 0.005 (10 pairs of comparison) was considered a significant threshold to control for multiple comparison phenomenon. We also conducted a sensitivity analysis by excluding bladder cancer cases and person‐years observed within the first two years of observation after the enrollment. The Schoenfeld residual test was used to test the proportionality assumption for hazards over follow‐up time and no violation was found. The linear trend for bladder cancer risk with LCD scores was tested based on the ordinal values of their quartiles. We also tested the interactions in the LCD score‐bladder cancer risk association between different groups of selected factors (i.e., sex, BMI, smoking status, alcohol drinking status and history of diabetes) by including the product term of LCD score and the selected risk factors in the multivariable Cox regression models.

All statistical analyses were performed using the SAS version 9.4 computer software (SAS Institute Inc., Cary, NC). All *P* values presented are two‐sided and *P‐*values less than 0.05 were statistically significant, except those with the Bonferroni correction method.

## 3 RESULTS

We identified 250 incident cases of bladder cancer among 61 321 study participants of the SCHS who were free of cancer at baseline after a mean (standard deviation) follow‐up of 17.6 (5.3) years (or 819 573 person‐years). The mean age (± SD) of cancer diagnosis was 60.8 (±7.9).

Compared to cohort participants who remained free of bladder cancer during the study period (aka non‐cases hereafter), individuals who developed incident bladder cancer (or cases) were older, more likely to be male and ever smokers, and less likely to have physical activity weekly (all *P*‐values <0.05). Patients with incident bladder cancer consumed less amount of vitamin A than non‐cases. The distributions of the level of education, dialect, coffee drinking status, BMI level, daily intakes of soy, fibre, total fat, animal fat, saturated fat, monounsaturated fat, polyunsaturated fat, total protein and total calorie intake were comparable between cases and non‐cases (Table 1).

**TABLE 1:** Distribution of baseline characteristics among study participants by bladder cancer status, the Singapore Chinese health study, 1993–2015.

Characteristics	Bladder cases (n = 250)	Non‐cases (n = 61 071)	*P‐value*
Age, (Mean±SD)	60.8 ± 7.9	56.4 ± 8.0	**<0.0001**
Sex			
Male	186 (74.4)	27 107 (44.4)	**<0.0001**
Female	64 (25.6)	33 964 (55.6)	
Highest level of education			
No formal education	55 (22.0)	16 606 (27.2)	0.07
Primary school	128 (51.2)	27 096 (44.4)	
Secondary school or higher	67 (26.8)	17 369 (28.4)	
Dialect			
Cantonese	102 (40.8)	28 223 (46.2)	0.09
Hokkien	148 (59.2)	32 848 (53.8)	
Weekly physical activity^a^			
No	152 (60.8)	40 931 (67.0)	**0.04**
Yes	98 (39.2)	20.140 (33.0)	
Smoking status			
Never smoker	106 (42.4)	42 477 (69.5)	<0.0001
Ever smoker	144 (57.6)	18 594 (30.4)	
Alcohol consumption			
Non‐Drinker/Monthly drinker	219 (87.6)	53 925 (88.3)	0.91
Weekly drinker	21 (8.4)	4997 (8.2)	
Daily drinker	10 (4.0)	2149 (3.5)	
Coffee drinking status			
Non‐drinker/monthly/weekly	66 (26.4)	18 083 (29.6)	0.67
1 cup/day	92 (36.8)	21 995 (36.0)	
2–3 cups/day	80 (32.0)	18 583 (30.4)	
≥ 4 cups/day	12 (4.8)	2410 (3.9)	
History of diabetes			
No	227 (90.8)	55 625 (91.1)	0.88
Yes	23 (9.2)	5446 (8.9)	
Family history of cancer			
No	246 (98.4)	59 726 (97.8)	0.67
Yes	4 (1.6)	1.345 (2.2)	
BMI, kg/m (Mean±SD)	22.8 ± 2.9	23.1 ± 3.3	0.09
Dietary Soy intake (g/1000 Kcal)	111.3 ± 100.2	111.8 ± 111.1	0.93
Total Energy Intake (kcal/day)	1573.8 ± 510.3	1556.6 ± 566.4	0.63
Carbohydrate 1 (gr/day) (Mean±SD)	230.1 ± 73.4	227.8 ± 81.1	0.66
Dietary fibre (gr/day) (Mean±SD)	12.1 ± 5.2	12.7 ± 5.8	0.12
Calcium (mg/1000 kcal) (Mean±SD)	412.1 ± 222.2	415.3 ± 229.5	0.83
Vitamin A (IU/1000 kcal) (Mean±SD)	4694.2 ± 2668.4	5111.6 ± 3353.9	0.05
Vitamin C (mg/1000 kcal) (Mean±SD)	100.4 ± 135.2	102.9 ± 134.2	0.77
Vitamin E (α‐tocopherol equivalents) (mg/1000 kcal) (Mean±SD)	11.7 ± 44.1	12.6 ± 47.3	0.76
Total fat (gr/day) (Mean±SD)	44.4 ± 20.0	44.1 ± 21.1	0.78
Animal fat (gr/day) (Mean±SD)	16.2 ± 9.8	15.2 ± 9.8	0.09
Plant fat (gr/day) (Mean±SD)	28.2 ± 12.6	28.9 ± 13.6	0.44
Saturated fat (gr/day) (Mean±SD)	15.9 ± 7.8	15.6 ± 8.1	0.56
Monunsaturated fat (gr/day) (Mean±SD)	15.1 ± 7.0	14.9 ± 7.2	0.64
Polyunsataturated fat (gr/day) (Mean±SD)	8.7 ± 4.5	8.9 ± 4.9	0.57
Total protein (gr/day) (Mean±SD)	60.1 ± 22.9	59.2 ± 59.0	0.56

^a^ Including strenuous physical activity and/or vigorous work.

* Fisher exact test.

Compared to those in the lowest quartile of total LCD score, participants with the highest quartile of total LCD score were younger, more likely to be female, having a higher level of education and a history of type 2 diabetes, but less likely to smoke cigarettes or drink alcohols. Additionally, they had higher intakes of calories, protein and fat but lower intakes of carbohydrate. Similar patterns were observed for animal‐based and plant‐based LCD scores (Table S2).

Although there was a marginal association between total LCD score and bladder cancer risk {HR = 1.15, 95% CI: 1.00–1.32; *P*_*trend*_ = 0.06), a statistically significant positive association between animal‐based LCD score and risk of bladder cancer was observed. Compared with the lowest quartile, the HRs (95% CIs) of bladder cancer for quartiles 2, 3, and 4 of the animal‐based LCD score were 1.09 (0.76–1.56), 1.17 (0.81–1.70) and 1.53 (1.09–2.17), respectively; (HR_per‐SD increment_ = 1.16, 95% CI: 1.02–1.32; *P*_*trend*_ = 0.01). No association between plant‐based LCD score and risk of bladder cancer (HR _per‐SD increment_ = 1.08, 95% CI: 0.91–1.28; *P*_*trend*_ = 0.78) (Table 2).

**TABLE 2:** Association between Low‐carbohydrate diet (LCD) score and risk of bladder cancer, the Singapore Chinese health study, 1993–2015.

LCD scores	Person‐year	# bladder cancer cases	HR^a^ (95% CI)
Overall LCD			
Q1 (lowest)	255 912	62	1.00
Q2	293 785	66	1.06 (0.74–1.50)
Q3	269 553	59	1.14 (0.79–1.65)
Q4 (highest)	263 833	63	1.43 (0.98–2.09)
*P*_*trend*_			0.06
Continuous scale (per SD increment)			1.15 (1.00–1.32)
Animal‐based LCD			
Q1 (lowest)	280 454	61	1.00
Q2	273 270	59	1.09 (0.76–1.56)
Q3	240 856	53	1.17 (0.81–1.70)
Q4 (highest)	288 504	77	1.53 (1.09–2.17)
*P*_*trend*_			0.01
Continuous scale (per SD increment)			1.16 (1.02–1.32)
Plant‐based LCD			
Q1 (lowest)	270 444	70	1.00
Q2	274 598	67	1.06 (0.75–1.51)
Q3	242 714	57	1.15 (0.78–1.70)
Q4 (highest)	295 336	56	1.04 (0.66–1.63)
*P*_*trend*_			0.78
Continuous scale (per SD increment)			1.08 (0.91–1.28)

^a^ Models adjusted for age, sex, dialect, year of enrollment, education level, smoking status (never smoker, ever smoker), coffee drinking status, alcohol drinking status (non‐drinker, monthly drinker, weekly drinker, daily drinker), total soy intake, total energy intake, BMI (<18.5, 18.5‐ < 23.0, 23.0‐ < 27.0, ≥27.0), physical activity and history of diabetes status.

Abbreviations: CI: confidence interval; HR: hazard ratio; LCD: low‐carbohydrate diet; SD: standard deviation.

In stratified analysis, we did not find an association between total LCD, animal‐based and plant‐based LCD scores with the risk of bladder cancer and there was no interaction between LCD with such factors (i.e., BMI, smoking status, alcohol drinking status and type 2 diabetes history) in the association with risk of bladder cancer (All *P's‐values* >0.05). (Table 3).

**TABLE 3:** Association between Low‐carbohydrate diet score and risk of bladder cancer from stratified analyses, the Singapore Chinese health study, 1993‐2015^a^.

Stratified by participant's characteristics		Q1	Q2	Q3	Q4	P_*Trend*_	Continuous scale (per SD increment)	*P*_*Interaction*_
**Overall LCD**								
Men	# Cases	50	49	44	43			0.43
	HR (95% CI)	1.00	0.96 (0.65–1.44)	1.05 (0.69–1.60)	1.23 (0.79–1.90)	0.34	1.09 (0.93–1.27)	
Women	# Cases	12	17	15	20			
	HR (95% CI)	1.00	1.43 (0.68–3.01)	1.49 (0.68–3.25)	2.27 (1.04–4.95)	0.05	1.36 (1.03–1.79)	
BMI < 23	# Cases	37	36	35	34			0.92
	HR (95% CI)	1.00	0.97 (0.61–1.54)	1.14 (0.71–1.84)	1.32 (0.8–2.19)	0.22	1.12 (0.94–1.34)	
BMI ≥ 23	# Cases	25	30	24	29			
	HR (95% CI)	1.00	1.2 (0.7–2.05)	1.13 (0.64–2.01)	1.57 (0.88–2.79)	0.17	1.18 (0.96–1.45)	
Never smokers	# Cases	28	28	23	27			
	HR (95% CI)	1.00	1.04 (0.61–1.77**)**	1.07 (0.61–1.9)	1.55 (0.87–2.76)	0.17	1.17 (0.95–1.45)	0.41
Ever smokers	# Cases	34	38	36	36			
	HR (95% CI)	1.00	1.08 (0.67–1.72)	1.21 (0.75–1.97)	1.38 (0.83–2.27)	0.18	1.14 (0.97–1.36)	
Non/monthly drinker	# Cases	56	59	49	55			0.70
	HR (95% CI)	1.00	1.09 (0.75–1.58)	1.09 (0.73–1.62)	1.42 (0.95–2.12)	0.11	1.13 (0.98–1.31)	
Weekly/daily drinker	# Cases	6	7	10	8			
	HR (95% CI)	1.00	0.86 (0.29–2.58)	1.49 (0.52–4.25)	1.57 (0.5–4.93)	0.27	1.28 (0.87–1.90)	
No diabetics history	# Cases	57	61	52	57			
	HR (95% CI)	1.00	1.09 (0.75–1.57)	1.13 (0.77–1.67)	1.51 (1.01–2.24)	0.05	1.17 (1.01–1.34)	0.62
Diabetes history	# Cases	5	5	7	6			
	HR (95% CI)	1.00	0.8 (0.23–2.78)	1.18 (0.36–3.83)	0.85 (0.24–3.07)	0.98	1.00 (0.64–1.56)	
**Animal‐based LCD**								
Men	# Cases	46	45	37	58	0.07		0.92
	HR (95% CI)	1.00	1.08 (0.71–1.63)	1.05 (0.68–1.62)	1.48 (0.99–2.19)		1.12 (0.96–1.30)	
Women	# Cases	15	14	16	19			
	HR (95% CI)	1.00	1.11 (0.53–2.3)	1.54 (0.75–1.35)	1.71 (0.84–3.45)	0.09	1.30 (1.00–1.68)	
BMI < 23	# Cases	35	36	28	43			0.65
	HR (95% CI)	1.00	1.13 (0.71–1.81)	1.06 (0.64–1.76)	1.45 (0.92–2.29)	0.14	1.11 (0.94–1.32)	
BMI ≥ 23	# Cases	26	23	25	34			
	HR (95% CI)	1.00	1.03 (0.59–1.81)	1.32 (0.76–2.31)	1.63 (0.96–2.75)	0.05	1.22 (1.01–1.49)	
Never smokers	# Cases	28	21	28	29			0.99
	HR (95% CI)	1.00	0.89 (0.5–1.57)	1.49 (0.88–2.54)	1.47 (0.86–2.51)	0.06	1.18 (0.97–1.44)	
Ever smokers	# Cases	33	38	25	48			
	HR (95% CI)	1.00	1.26 (0.79–2.02)	0.97 (0.57–1.63)	1.61 (1.02–2.53)	0.08	1.16 (0.98–1.37)	
Non/monthly drinker	# Cases	57	53	45	64			0.56
	HR (95% CI)	1.00	1.09 (0.75–1.59)	1.12 (0.76–1.67)	1.46 (1.01–2.11)	0.05	1.13 (0.99–1.29)	
Weekly/daily drinker	# Cases	4	6	8	13			
	HR (95% CI)	1.00	1.16 (0.33–4.12)	1.68 (0.50–5.64)	2.21 (0.71–6.91)	0.11	1.41 (0.97–2.05)	
No diabetics history	# Cases	57	52	48	70			0.83
	HR (95% CI)	1.00	1.04 (0.71–1.52**)**	1.14 (0.78–1.68)	1.53 (1.07–2.19)	0.02	1.16 (1.02–1.33)	
Diabetes history	# Cases	4	7	5	7			
	HR (95% CI)	1.00	1.87 (0.54–6.50)	1.62 (0.43–6.13)	1.7 (0.48–6.08)	0.52	1.16 (0.76–1.78)	
**Plant‐based LCD**								
Men	# Cases	55	49	44	38			
	HR (95% CI)	1.00	0.96 (0.64–1.43)	1.09 (0.69–1.71)	0.85 (0.50–1.45)	0.72	1.02 (0.84–1.24)	0.96
Women	# Cases	15	18	13	18			
	HR (95% CI)	1.00	1.39 (0.69–2.82)	1.28 (0.57–2.86)	1.71 (0.71–4.08)	0.29	1.25 (0.90–1.74)	
BMI < 23	# Cases	44	36	34	28			
	HR (95% CI)	1.00	0.88 (0.56–1.40)	1.05 (0.63–1.74)	0.78 (0.42–1.43)	0.58	1.04 (0.83–1.30)	0.50
BMI ≥ 23	# Cases	26	31	23	28			
	HR (95% CI)	1.00	1.36 (0.79–2.33)	1.29 (0.69–2.39)	1.48 (0.75–2.91)	0.31	0.92 (0.73–1.15)	
Never smokers	# Cases	32	33	18	13			0.03
	HR (95% CI)	1.00	1.19 (0.72–1.96**)**	0.84 (0.45–1.58)	1.07 (0.54–2.12)	0.90	1.04 (0.80–1.34)	
Ever smokers	# Cases	38	34	39	33			
	HR (95% CI)	1.00	0.97 (0.6–1.57)	1.37 (0.82–2.29)	1.05 (0.58–1.90)	0.60	1.12 (0.90–1.40)	
Non/monthly drinker	# Cases	62	60	49	48			0.55
	HR (95% CI)	1.00	1.11 (0.77–1.61)	1.18 (0.78–1.80)	1.08 (0.66–1.74)	0.69	1.10 (0.92–1.31)	
Weekly/daily drinker	# Cases	8	7	8	8			
	HR (95% CI)	1.00	0.75 (0.26–2.12)	0.96 (0.32–2.86)	0.78 (0.22–2.81)	0.81	0.97 (0.60–1.56)	
No diabetics history	# Cases	64	61	52	50			0.59
	HR (95% CI)	1.00	1.1 (0.76–1.58)	1.21 (0.8–1.83)	1.11 (0.69–1.78)	0.56	1.11 (0.93–1.32)	
Diabetes history	# Cases	6	6	5	6			
	HR (95% CI)	1.00	0.74 (0.23–2.43)_	0.69 (0.18–2.57)	0.5 (0.12–2.16)	0.37	0.83 (0.48–1.44)	

^a^ Models adjusted for age, sex, dialect, year of enrollment, education level, smoking status (never smoker, ever smoker), coffee drinking status, alcohol drinking status (Non‐drinker, monthly drinker, weekly drinker, daily drinker), total energy intake, total soy intake, BMI (<18.5, 18.5‐ < 23.0, 23.0‐ < 27.0, ≥27.0), physical activity and history of diabetes status (if possible).

Abbreviations: CI: confidence interval; HR: hazard ratio; LCD: low‐carbohydrate diet; SD: standard deviation.

In the sensitivity analysis on the entire cohort after excluding bladder cancer cases and person‐years within the first two years of observation, the association between animal‐based LCD score and risk of bladder cancer remained. Accordingly, compared to the lowest quartile, the HRs of bladder cancer for the highest quartiles of total LCD, animal‐based LCD and plant‐based LCD scores, in comparison to respective lowest quartiles, were 1.33 (95% CI: 0.89–1.98, *P*_*trend*_ = 0.18), 1.45 (95% CI: 1.01–2.08, *P*_*trend*_ = 0.03) and 1.11 (95% CI: 0.63–1.62, *P*_trend_ = 0.87), respectively (Table 4 **)**. The respective HRs (95% CIs) per SD of total LCD, animal‐based LCD and plant‐based LCD scores were 1.13 (0.98–1.30), 1.14 (1.00–1.30) and 1.06 (0.89–1.27).

**TABLE 4:** Association between low‐carbohydrate diet (LCD) score and risk of bladder cancer after excluding incident cases and person‐years in the first two years post‐enrollment, the Singapore Chinese health study, 1993–2015.

Characteristics	Person‐year	# bladder cancer cases	HR^a^ (95% CI)
Overall LCD			
Q1	226 646	58	1.00
Q2	260 726	63	1.07 (0.75–1.54)
Q3	239 540	54	1.11 (0.76–1.63)
Q4	234 468	55	1.33 (0.89–1.98)
*P*_*trend*_			0.18
Continuous scale (per SD increment)			1.13 (0.98–1.30)
Animal‐based LCD			
Q1	248 664	57	1.00
Q2	242 597	54	1.07 (0.73–1.55)
Q3	214 030	51	1.20 (0.82–1.76)
Q4	256 089	68	1.45 (1.01–2.08)
*P*_*trend*_			0.03
Continuous scale (per SD increment)			1.14 (1.00–1.30)
Plant‐based LCD			
Q1	239 226	64	1.00
Q2	243 688	63	1.09 (0.76–1.56)
Q3	215 653	53	1.16 (0.77–1.75)
Q4	262 813	50	1.01 (0.63–1.62)
*P*_*trend*_			0.87
Continuous scale (per SD increment)			1.06 (0.89–1.27)

^a^ Models adjusted for age, sex, dialect, year of enrollment, education level, smoking status (never smoker, ever smoker), coffee drinking status, alcohol drinking status (non‐drinker, monthly drinker, weekly drinker, daily drinker), total energy intake, total soy intake, BMI (<18.5, 18.5‐ < 23.0, 23.0‐ < 27.0, ≥27.0), physical activity and history of diabetes status.

Abbreviations: CI: confidence interval; HR: hazard ratio; LCD: low‐carbohydrate diet; SD: standard deviation.

## 4 DISCUSSION

The present study of more than 63 000 participants from the Singapore Chinese Health Study shows a statistically significant, positive association between animal‐based LCD score and risk of bladder cancer. We did not find a significant association between plant‐based LCD score and risk of bladder cancer.

To our knowledge, the current analysis was the first study evaluating the association between low‐carbohydrate diet scores and risk of bladder cancer in the Chinese population. Only one study examined the association between LCD scores and risk of bladder cancer incidence in Asian population. Accordingly, a recent report by Cai et al.^11^ from the Japan Public Health Center (JPHC)‐based Prospective Study Group, comprising 90 171 Japanese and 247 incident cases of bladder cancer with a median follow‐up of 17.0 years, found null associations between total (HR_Q5 vs Q1_ = 1.00, 95% CI: 0.72–1.39, *P*_*trend*_ = 0.81), animal‐based (HR_Q5 vs Q1_ = 1.03, 95% CI: 0.75–1.41, *P*_*trend*_ = 0.81) and plant‐based‐LCD (HR_Q5 vs Q1_ = 0.78, 95% CI: 0.58–1.07, *P*_*trend*_ = 0.15) scores with risk of bladder cancer. With a comparable sample size (n = 250 incident cases of bladder cancer in ours) and study design, we found a statistically significant, positive association between animal‐based LCD score and risk of bladder cancer in this prospective cohort of Chinese Singaporeans. The discrepancy between our results and those in the JPHC cohort study might be due to differences in dietary patterns and lifestyle's characteristics between Chinese Singaporeans and the Japanese population. For instance, the Chinese Singaporeans have higher consumption of animal protein (Mean intake‐Quintile 5: 45 g/day) and carbohydrate (Mean intake‐Quintile 1: 250 g/day) than those in the Japanese population (Mean intake‐Quintile 5 = 11.3 g/day and Quintile 1 = 66 g/day, respectively).

Our findings of a positive association for animal‐based LCD with risk of bladder cancer may be related to a diet with high protein and fat from animal sources, but low total carbohydrate. Higher intake of fat and protein was found to be associated with an elevated risk of bladder cancer in a previous meta‐analysis that included five prospective cohort studies and eight case–control studies of bladder cancer.^27^ The pooled risk estimate per 100 g of daily red meat intake was 1.51 (95% CI: 1.13–2.02) derived from the case–control studies but not cohort studies. The same meta‐analysis also found that the pooled risk estimate per 50 g of daily processed meat intake was 1.20 (95% CI: 1.06–1.37). These are also biologically plausible because carcinogens such as heterocyclic amines (HCAs), polycyclic aromatic hydrocarbons (PAHs) or N‐nitroso compounds (NOCs) generated during the cooking and/or processing processes are pro‐oxidants, leading to cancer initiation and development.^28^, ^29^, ^30^ Processed meat and processed red meat were also classified as Group 1 carcinogens (i.e., carcinogenic to human) and red meat as Group 2A carcinogens (i.e. probably carcinogenic to human) by the International Agency for Research on Cancer (IARC).^31^

Carbohydrate sources in the plant‐based LCD score were from vegetables, fruits or legumes, which provide extensive sources of prevention potentials, including antioxidants.^32^ In a recent pooled analysis of 11 case–control studies in the BLadder cancer Epidemiology and Nutritional Determinants (BLEND) consortium (i.e., 5637 bladder cancer cases and 10 504 controls), Boot et al.^33^ showed that both fruits and vegetables were associated with reduced risk of bladder cancer. We did not find an association between plant‐based LCD and bladder cancer risk in the current analysis.

Our study has several limitations. It is not possible to evaluate the changes in diet over time as we collected dietary information at the baseline only. This might result in the non‐differential misclassification phenomenon and change the overall association toward the null.^34^ We also were not able to construct two important scores that reflect the carbohydrate intakes, the glycemic index and glycemic load,^35^, ^36^, ^37^ and evaluate their associations with bladder cancer risk in the current analysis. It is noted that a recent meta‐analysis, including five cohort studies and seven case–control studies, found a dose–response positive association between glycemic index and risk of bladder cancer.^38^ The reason for not constructing these two indices is that there is difference in diets between Chinese and Westerners and most of the components used to construct these two scores were derived from Western population‐based studies. Findings from the current analysis are not generalizable to other populations, including those in the Western populations due to the differences in diets such as higher level of carbohydrate and vegetable intakes and lower level of protein/fat intake from animal sources in the Chinese population.^39^ In addition, residual confounding effects are inescapable in our study, similar to analysis of other observational studies, despite the fact that we tried to control such effects in multivariable regression models. Also, in the stratified analysis for the purpose of hypothesis generation, we did not adjust for multiple comparisons; therefore, our results in the stratified analysis should be interpreted cautiously. Finally, information on tumour stage or grade is not available in the cancer registry linkage, we were unable to conduct further stratified analysis, including the status of the tumour rarely lethal versus highly lethal.

However, our study also has several strengths. This analysis, to our knowledge, might be the first attempt to examine the association between LCD scores and the risk of bladder cancer in Chinese population. Also, because of the prospective cohort study design, it allowed us to obtain information on diet, lifestyle and other risk factors prior the diagnosis of bladder cancer and minimize the potential effects of symptoms and diagnosis of the disease outcome on patients' diets, habits and lifestyles. The prospective cohort study design also allowed us to follow up study participants for a long period of time and to have sufficient power to determine the prolonged effect of LCD pattern on the risk of bladder cancer development. Lastly, a comprehensive questionnaire employing in the SCHS allowed us to assess different lifestyles and medical history and use them in the multivariable regression models to control for potential confounding effects of such modifiable factors on the observed association between animal‐based LCD score and bladder cancer risk.

In summary, the current analysis of data from SCHS shows a statistically significant, positive association between animal‐based LCD score and risk of developing bladder cancer in a prospective cohort of more than 61 000 Chinese in Singapore. The findings suggest that dietary modification with more plant food and less animal food may be a potential strategy for primary prevention against the development of bladder cancer.

## AUTHOR CONTRIBUTIONS

**Yen Thi‐Hai Pham:** Conceptualization; methodology; investigation; formal analysis; validation; writing—original draft; writing—review and editing. **Renwei Wang:** Data curation; methodology; investigation; formal analysis; validation; writing—review and editing. **Jian‐Min Yuan:** Conceptualization; methodology; investigation; formal analysis; supervision; data curation; validation; writing—review and editing. **Hung N. Luu:** Conceptualization; methodology; investigation; formal analysis; supervision; data curation; validation; writing—original draft; writing—review and editing.

## CONFLICT OF INTEREST STATEMENT

None to declare.

## Supporting information


**Table S1.** Cutoff Values of Daily Calorie (%) from Total Fat, Protein and Carbohydrate for Individual Low‐carbohydrate Diet (LCD) Scores, the Singapore Chinese Health Study, 1993–2015.
**Table S2.** Baseline characteristics and daily intake of nutrient by participants in the highest compared with the lowest quartiles of total, animal‐based and plant‐based low‐carbohydrate diet scores (LCDs). The Singapore Chinese Health Study, 1993–2015.

## Data Availability

The data that support the findings of this study are available on request from the corresponding author. The data are not publicly available due to privacy or ethical restrictions.
