# Supplementary material for: Low‐carbohydrate diet score and risk of bladder cancer: Findings from a prospective cohort study
Source: BJUI Compass. 2025 Jun 2;6(6):e70033. doi: 10.1002/bco2.70033 (PMC12129589; doi:10.1002/bco2.70033)
Supplement: Supplementary file 1 — Table S1. Cutoff Values of Daily Calorie (%) from Total Fat, Protein and Carbohydrate for Individual Low‐carbohydrate Diet (LCD) Scores, the Singapore Chinese Health Study, 1993–2015. Table S2. Baseline characteristics and daily intake of nutrient by participants in the highest compared with the lowest quartiles of total, animal‐based and plant‐based low‐carbohydrate diet scores (LCDs). The Singapore Chinese Health Study, 1993–2015. [file BCO2-6-e70033-s001.docx]

**Supplementary Table 1.** **Cutoff Values of Daily Calorie (%) from Total Fat, Protein and Carbohydrate for Individual Low-carbohydrate Diet (LCD) Scores,**

**the Singapore Chinese Health Study, 1993-2015**

| **LCD Score** | **Total Fat**  **(% energy)** | **Total Protein**  **(% energy)** | **Total Carbohydrate**  **(% energy)** |
| --- | --- | --- | --- |
| 0 | ≤17.46 | ≤11.95 | ≥69.02 |
| 1 | 17.46-19.94 | 11.95-12.94 | 65.83-69.02 |
| 2 | 19.94-21.67 | 12.94-13.66 | 63.57-65.83 |
| 3 | 21.67-23.16 | 13.66-14.27 | 61.67-63.57 |
| 4 | 23.16-24.48 | 14.27-14.84 | 59.94-61.67 |
| 5 | 24.48-25.77 | 14.84-15.41 | 58.24-59.94 |
| 6 | 25.77-27.11 | 15.41-15.99 | 56.52-58.24 |
| 7 | 27.11-28.58 | 15.99-16.62 | 54.64-56.52 |
| 8 | 28.58-30.26 | 16.62-17.38 | 52.41-54.64 |
| 9 | 30.26-32.63 | 17.38-18.52 | 49.26-52.41 |
| 10 | ≥32.63 | ≥18.52 | ≤49.26 |

**Supplementary Table 2. Baseline characteristics and daily intake of nutrient by participants in the highest compared with the lowest quartiles of total, animal-based, and plant-based low-carbohydrate diet scores (LCDs)**

**The Singapore Chinese Health Study, 1993-2015**

|  | **Total LCD** | | | **Animal-based LCD** | | | **Plant-based LCD** | | |
| --- | --- | --- | --- | --- | --- | --- | --- | --- | --- |
|  | **Quartile 1**  **N, %** | **Quartile 4**  **N, %** | ***P-value*** | **Quartile 1**  **N, %** | **Quartile 4**  **N, %** | ***P-value*** | **Quartile 1**  **N, %** | **Quartile 4**  **N, %** | ***P-value*** |
| Age, (Mean±SD) | 57.9 ± 8.1 | 55.0 ± 7.7 | <0.001 | 57.8 ± 8.1 | 55.2 ± 7.8 | <0.001 | 58.0 ± 8.1 | 55.0 ± 7.7 | <0.001 |
| Sex |  |  |  |  |  |  |  |  |  |
| Female | 7,377 (50.0) | 9,056 (61.2) | <0.001 | 8,872 (55.4) | 8,983 (55.0) | 0.15 | 7,752 (49.2) | 10,243 (62.6) | <0.001 |
| Male | 7,382 (50.0) | 5,731 (38.8) |  | 7,150 (44.6) | 7,351 (45.0) |  | 8,008 (50.8) | 6,116 (37.4) |  |
| Highest Level of Education |  |  |  |  |  |  |  |  |  |
| No formal education | 4,735 (32.1) | 3,446 (23.3) | <0.001 | 5,238 (32.7) | 3,708 (22.7) | <0.001 | 5,248 (33.3) | 3,679 (22.5) | <0.001 |
| Primary school | 6,766 (45.8) | 6,228 (42.1) |  | 7,129 (44.5) | 7,119 (43.6) |  | 7,273 (46.2) | 6,784 (41.5) |  |
| Secondary school or higher | 3,258 (22.1) | 5,113 (34.6) |  | 3,655 (22.8) | 5,507 (33.7) |  | 3,239 (20.5) | 5,896 (36.0) |  |
| Dialect |  |  |  |  |  |  |  |  |  |
| Cantonese | 6,910 (46.8) | 6,890 (46.6) | 0.07 | 7,345 (45.8) | 7,623 (46.7) | 0.49 | 7,024 (44.6) | 7,854 (48.0) | <0.001 |
| Hokkien | 7,849 (53.2) | 7,897 (53.4) |  | 8,677 (54.2) | 8,711 (53.3) |  | 8,736 (55.4) | 8,505 (52.0) |  |
| Weekly Physical Activity |  |  |  |  |  |  |  |  |  |
| No | 10,013 (67.8) | 9,983 (67.5) | 0.006 | 10,903 (68.0) | 11,056 (67.7) | <0.001 | 10,841 (68.8) | 10,748 (65.7) | <0.001 |
| Yes | 4,746 (32.2) | 4,804 (32.5) |  | 5,119 (32.0) | 5,278 (32.3) |  | 4,919 (31.2) | 5,611 (34.3) |  |
| Smoking Status |  |  |  |  |  |  |  |  |  |
| Never Smoker | 9,694 (65.7) | 10,829 (73.2) | <0.001 | 11,182 (69.8) | 11,166 (68.4) | 0.004 | 10,020 (63.6) | 12,319 (75.3) | <0.001 |
| Ever Smoker | 5,065 (34.3) | 3,958 (26.8) |  | 4,840 (30.2) | 5,168 (31.6) |  | 5,740 (36.4) | 4,040 (24.7) |  |
| Alcohol Consumption |  |  |  |  |  |  |  |  |  |
| Non-Drinker/Monthly drinker | 13,259 (89.8) | 13,212 (89.9) | <0.001 | 14,717 (91.8) | 14,057 (86.1) | <0.001 | 14,113 (89.5) | 14,454 (89.4) | <0.001 |
| Weekly drinker | 1,096 (7.4) | 1,225 (8.3) |  | 984 (6.1) | 1,626 (9.9) |  | 1,194 (7.6) | 1,385 (8.5) |  |
| Daily drinker | 404 (2.7) | 350 (2.4) |  | 321 (2.0) | 651 (4.0) |  | 453 (2.9) | 520 (3.2) |  |
| Coffee drinking status |  |  |  |  |  |  |  |  |  |
| Non-drinker/monthly/weekly | 4,224 (28.8) | 4,711 (31.9) | <0.001 | 4,713 (39.4) | 5,034 (30.8) | 0.01 | 4,537 (28.8) | 5243 (32.0) | <0.001 |
| 1 cup/day | 5,121 (34.7) | 5,565 (37.6) |  | 5,824 (36.3) | 5,801 (35.5) |  | 5,300 (33.6) | 6199 (37.9) |  |
| 2-3 cups/day | 4,780 (33.0) | 4,013 (27.1) |  | 4,874 (30.4) | 4,836 (29.6) |  | 5,189 (32.9) | 4418 (27.0) |  |
| ≥4 cups/day | 634 (4.3) | 498 (3.4) |  | 611 (3.8) | 663 (4.1) |  | 734 (4.7) | 499 (3.0) |  |
| History of Diabetes |  |  |  |  |  |  |  |  |  |
| No | 13,717 (92.9) | 13,111 (88.7) | <0.001 | 14,773 (92.2) | 14,644 (89.7) | <0.001 | 14,587 (92.6) | 14,667 (89.7) | <0.001 |
| Yes | 1,042 (7.1) | 1,676 (11.3) |  | 1,249 (7.8) | 1,690 (10.3) |  | 1,173 (7.4) | 1,692 (10.3) |  |
| Family History of cancer |  |  |  |  |  |  |  |  |  |
| No | 13,080 (88.6) | 12,458 (84.2) | <0.001 | 14,133 (88.2) | 13,796 (84.5) | <0.001 | 13,975 (88.7) | 13,723 (83.9) | <0.001 |
| Yes | 1679 (11.4) | 2,329 (15.7) |  | 1,880 (11.8) | 2,538 (15.5) |  | 1,785 (11.3) | 2,636 (16.1) |  |
| BMI, kg/m (Mean±SD) | 23.0±3.2 | 23.3±3.4 | <0.001 | 23.1±3.2 | 23.2±3.3 | 0.009 | 23.0±3.2 | 23.3±3.3 | <0.001 |
| Total Energy Intake (kcal/day) (Mean±SD) | 1,466.5±501.8 | 1,685.4±632.1 | <0.001 | 1,433.7±485.5 | 1,705.0±640.0 | <0.001 | 1,464.4±508.8 | 1,668.9±619.0 | <0.001 |
| Carbohydrate (gr/day) (Mean±SD) | 250.2±84.3 | 209.9±77.0 | <0.001 | 241.2±82.2 | 216.9±79.9 | <0.001 | 242.4±84.0 | 216.6±78.2 | <0.001 |
| Dietary fiber (gr/day) (Mean±SD) | 11.4±5.4 | 13.8±6.2 | <0.001 | 12.2±5.8 | 13.0±5.9 | <0.001 | 10.7±5.0 | 14.8±6.4 | <0.001 |
| Total fat (gr/day) (Mean±SD) | 30.0±11.8 | 59.9±25.0 | <0.001 | 31.1±12.8 | 58.3±25.0 | <0.001 | 31.7±13.3 | 57.1±24.2 | <0.001 |
| Animal fat (gr/day) (Mean±SD) | 9.3±5.5 | 21.8±12.0 | <0.001 | 8.0±4.3 | 23.6±11.5 | <0.001 | 11.8±7.3 | 18.0±10.9 | <0.001 |
| Plant fat (gr/day) (Mean±SD) | 20.6±8.3 | 38.1±16.0 | <0.001 | 23.1±10.4 | 34.7±15.7 | <0.001 | 19.9±7.8 | 39.1±15.8 | <0.001 |
| Saturated fat (gr/day) (Mean±SD) | 10.5±4.6 | 21.2±9.6 | <0.001 | 10.6±4.8 | 21.1±9.6 | <0.001 | 11.9±5.6 | 19.2±9.4 | <0.001 |
| Monunsaturated fat (gr/day) (Mean±SD) | 10.0±4.1 | 20.4±8.8 | <0.001 | 10.3±4.3 | 20.1±8.8 | <0.001 | 10.9±4.8 | 19.0±8.5 | <0.001 |
| Polyunsataturated fat (gr/day) (Mean±SD) | 6.2±3.0 | 11.9±6.0 | <0.001 | 6.8±3.6 | 11.2±5.8 | <0.001 | 5.6±2.4 | 12.6±5.9 | <0.001 |
| Total Protein (gr/day) (Mean±SD) | 45.7±16.5 | 75.5±28.1 | <0.001 | 45.2±16.1 | 75.0±28.0 | <0.001 | 48.5±17.8 | 71.1±27.5 | <0.001 |
| Animal protein (gr/day) (Mean±SD) | 19.8±9.3 | 45.9±18.9 | <0.001 | 18.5±8.2 | 47.0±18.1 | <0.001 | 24.3±11.6 | 39.2±18.8 | <0.001 |
| Plant protein (gr/day) (Mean±SD) | 7.3±4.1 | 15.8±8.2 | <0.001 | 8.7±5.6 | 13.7±7.5 | <0.001 | 6.2±3.0 | 17.6±8.0 | <0.001 |

Means and standard deviation are calculated for continuous variables. Weekly physical activity including strenuous physical activity and/or vigorous work

Abbreviations: BMI: body mass index; LCD: low-carbohydrate diet; SD: standard deviation.
